# Effect of Dietary Supplementation of Fermented Pine Needle Extract on Productive Performance, Egg Quality, and Serum Lipid Parameters in Laying Hens

**DOI:** 10.3390/ani11051475

**Published:** 2021-05-20

**Authors:** Damini Kothari, Jong-Seok Oh, Ju-Hee Kim, Woo-Do Lee, Soo-Ki Kim

**Affiliations:** Department of Animal Science and Technology, Konkuk University, Seoul 05029, Korea; damini.kth@gmail.com (D.K.); fafafarm@naver.com (J.-S.O.); ahdzm12@naver.com (J.-H.K.); caw147@naver.com (W.-D.L.)

**Keywords:** laying hen, egg production, antioxidant capacity, phytogenic feed additive

## Abstract

**Simple Summary:**

In recent decades, the use of phytogenic feed additives as natural growth promoters has been substantially increased in the poultry industry. Fermented pine (*Pinus densiflora*) needle drink is used as a functional beverage in Korea due to its antioxidant effects. Therefore, the aim of the current study is to determine the effects of fermented pine needle extract on laying performance, egg quality, lipid parameters, and lipid oxidation of eggs in laying hens. Supplementation of fermented pine needle extract in laying hens’ diet improved productive performance and egg quality parameters.

**Abstract:**

This study aimed to investigate the supplemental effects of fermented pine (*Pinus densiflora*) needle extract (FPNE) in laying hen diets on productive performance, egg quality, and serum lipid metabolites. A total of 108 40-week-old Hy-line brown laying hens were randomly assigned to one of the three dietary treatment groups: (1) basal diet + 0 mL FPNE/kg diet (CON), (2) basal diet + 2.5 mL FPNE/kg diet (T1), or (3) basal diet + 5 mL FPNE/kg diet (T2) for 6 weeks. Each group consisted of four replicates of nine hens each. Feed and water provided ad libitum. Results showed that dietary supplementation of FPNE increased egg production percentage (linear, *p* < 0.01 and quadratic, *p* < 0.05), egg mass (linear, *p* < 0.05), and feed intake (linear, *p* < 0.05) during the entire experimental period. In addition, dietary inclusion of FPNE significantly increased the eggshell color (linear, *p* < 0.01), egg yolk color (quadratic, *p* < 0.01), and eggshell breaking strength (linear, *p* < 0.05 and quadratic, *p* < 0.05) while the Haugh unit decreased (quadratic, *p* < 0.05). However, serum lipid profile did not differ among the dietary treatments (*p* > 0.05). Notably, antioxidant activity of egg yolk was improved by significantly decreasing the malondialdehyde content in egg yolks after 6 weeks of storage (linear, *p* < 0.001 and quadratic, *p* < 0.05). In summary, dietary inclusion of FPNE could improve laying performance and the antioxidant capacity of eggs.

## 1. Introduction

In the past two decades, the increasing consumer demand for poultry products from hens raised without antibiotics has intensified the research on the development of antibiotic alternatives to maintain or improve poultry health and performance [1,2]. In this context, phytogenic feed additives (PFA), plant-based extracts containing various bioactive compounds such as polyphenols, organic acids, essential oils, terpenoids, and aldehydes, have gained significant attention, and are reported to have beneficial effects on animal production and health [3,4]. The antiviral, antimicrobial, antioxidant, and anti-inflammatory properties of PFA are thought to be their primary modes of action [5]. For example, *Pinus densiflora* of the family Pinaceae, commonly known as Korean Red Pine, has been a potential source of bioactive components. *P. densiflora* has the wide natural distribution area in Japan, Korea, North-Eastern China, and the extreme South-East of Russia. Pine needles contain several bioactive components such as α-pinene, caryophyllene, beta-pinene and bisbenzene, camphene, borneol, phellandrene, quercetin, kaempferol, and terpene, which have been reported to have antimicrobial, antimutagenic, and antioxidant effects [6,7,8]. Pine needles also contain calcium (28 mg/100 g) and various amino acids such as glutamic acid, phenylalanine, leucine, and lysine, as well as vitamins such as niacin, riboflavin, beta carotene, and thiamine [9,10]. Despite a rich source of bioactive compounds, some researchers indicated that high content of condensed tannins in pine needles might affect nutrient absorption by reducing protein digestibility in animals [11,12]. In this context, fermentation has been reported as an effective process to remove tannins and thereby improving the nutritional quality of pine needles [13].

Fermented pine needle drink has been used as a functional beverage due to its various biological properties in Asia–Pacific regions especially in Taiwan, China, and Korea [13]. Self-fermented pine (*P. densiflora*) needle extract has been reported to contain phenolics, flavonoids, essential oils, and terpenoids, which might act as nourishing agents [13,14]. In an in vivo study, when self-fermented pine (*P. densiflora*) needle extract aged for 7 years (0.5 mL/day) was orally gavage in cholesterol-fed rats for 4 weeks, it reduced the blood plasma cholesterol and triglycerides [15]. In addition, the same extract (200 µg/mL) also inhibited the frequency and amplitude of pacemaker currents in interstitial cells of Cajal of the murine small intestine via ATP-sensitive potassium channels, which suggests the regulatory role of self-fermented pine needle extract in gastrointestinal motility [15]. Gastrointestinal motility is crucial for ensuring the proper transportation of ingested food and absorption of nutrients along the gut [16]. Chen et al. [17] demonstrated that fermented pine (*P. morrisonicola*) needle preparations (vinegar and alcohol) have better antioxidant activity than non-fermented products. Recently, Chiu et al. [13] demonstrated antioxidant and anti-inflammatory activities of ethyl acetate extract of fermented pine (*P. morrisonicola*) needles in lipopolysaccharide-treated RAW 264.7 macrophage cells. These effects were mediated through the modulation of NF-κB signaling pathway. The same authors also observed higher contents of phenolics and flavonoids in the fermented extract as compared with the non-fermented one, which might be due to various enzymatic reactions of microorganisms. Only one study in broilers has demonstrated that the fermented pine needle powder significantly improved the antioxidant status of birds [12]. However, literature investigating the effect of fermented pine needle extract (FPNE) in laying hens’ diet is very scarce. Therefore, the aim of this study is to evaluate the effect of dietary supplementation of FPNE on laying productivity, egg quality, serum lipid parameters, and antioxidant capacity of eggs.

## 2. Materials and Methods

### 2.1. Pine Needle Fermentation

Pine needles used in this experiment were collected from *Pinus densiflora*, a native to Bonghwa, Gyeongbuk, Korea. The collected needles were washed, air-dried, and stored at 4 °C until fermentation. The pine needles were analyzed for their nutrient composition using AOAC procedures [18] (Table 1). For fermentation, the pine needles were chopped to 1 cm in size and mixed with water and sugar in a ratio of 1:1:0.6 on weight basis and then spontaneously fermented in an earthen pot (120 L) with the covered lid at room temperature for 6 months. The pine needles were then removed, and the remaining solution was aged in the same pot at room temperature for another 6 months. After aging, this solution was termed as a fermented pine needle extract (FPNE). The FPNE was then transferred to a stainless-steel beverage tank and stored at 4–8 °C until further use. 

### 2.2. Characterization of FPNE

The pH of the FPNE was directly measured using a digital pH meter (iSTEC 735P, Daejeon, Korea). The total polyphenols, total flavonoids, and antioxidant activity of FPNE was measured. To measure the total phenol content, 0.1 mL of the extract was mixed with 2 mL 2% (*w*/*v*) Na_2_CO_3_ and vortexed for 3 min. Thereafter, 0.1 mL of 1N Folin–Ciocalteu reagent was added to the mixture and absorbance was measured at 700 nm after 30 min of incubation against a water blank. The total flavonoid content was measured using the aluminum chloride colorimetric method. Briefly, 0.5 mL of the extract was mixed with 1.5 mL 95% ethanol, followed by the addition of 0.1 mL of 10% (*w*/*v*) aluminum chloride, 0.1 mL of 1 M potassium acetate, and 2.8 mL of distilled water. After 30 min, the absorbance was measured at 415 nm against a water blank. The total polyphenol and flavonoid content were expressed as microgram of quercetin equivalent (QE) per ml of the extract. The antioxidant activity of FPNE was determined by measuring the 2,2-diphenyl-1-picrylhydrazyl (DPPH) radical scavenging ability. Briefly, 32 µL of the extract, 128 µL of distilled water, and 640 µL of 0.15 mM DPPH solution (Sigma Co., St. Louis, MO, USA) were added and allowed to react in the dark for 15 min at room temperature. The absorbance was measured at 515 nm against a water blank and the results were expressed as percent inhibition using the Equation (1):

Radical scavenging activity (%) = (A_c_ − A_s_)/A_c_ × 100, (1)

A_c_: Absorbance of control and A_s_: Absorbance of sample.

### 2.3. Experimental Animal and Design

A total of 108 Hy-Line brown laying hens (40 week of age) randomly allotted to three experimental groups with four replicates of nine birds each. Hens were housed nine per cage in a double-tier metal-cage system (90 cm wide, 90 cm high and 735 cm^2^ area) at a controlled temperature of 22 ± 2 °C. A corn-soybean meal basal diet was formulated to meet the nutritional requirement of laying hens [19] (Table 2).

The amount of FPNE added to the basal diet was 0 mL/kg (CON), 2.5 mL/kg (T1), and 5 mL/kg (T2). The appropriate amount of FPNE was added to the basal diet and mixed for 5 min using a feed mixer (DKM350SU, Daekwang Co., Ltd., Hwaseong, Korea). The experimental period lasted for 6 weeks after 2 weekadaptation on the basal diet. Feed in mash form and water were provided ad libitum. The photoperiod was set at 16 h of light and 8 h of darkness (16L:8D) throughout the study.

### 2.4. Laying Performance

Eggs from individual cages were weighed and recorded daily. Egg production was calculated as the rate of egg production (including normal and broken eggs) per hen per day. The average weight of the eggs was determined by measuring the weight of all the eggs laid daily. Egg mass was calculated by multiplying the egg production rate by average egg weight. Average daily feed intake was recorded weekly as the difference between the feed offered and the refusals during a week. Feed conversion ratio was calculated by dividing feed intake by egg mass.

### 2.5. Egg Quality Analysis

Every week, 3 eggs per replicate from each treatment group were randomly selected for analyses. Eggs were weighed individually, and eggshell strength was measured using an eggshell strength tester (Fugihira Industry Co., Ltd., Tokyo, Japan). Then the egg was cracked, and egg contents were placed on a glass plate, and the height of albumen was measured to calculate the Haugh unit (HU) as described by Haugh [20], using the formula: HU  =  100 × log (H  +  7.57 − 1.7 × W^0.37^), where H is the albumen height, and W is the egg weight. Color of fresh yolk was measured using the Roche yolk color fan (F. Hoffmann-La Roche Ltd., Basel, Switzerland). Eggshell color was measured by comparing with eggshell color fan (Eggshell color fan, Samyang, Korea). For eggshell thickness, the thickness of the eggshell was measured using a digital micrometer (Digimatic Micrometer, Series 293–330, Mitutoyo, Japan).

### 2.6. Serum Lipid Parameters 

At the end of experiment, after fasting for 6 h, blood samples of eight laying hens per treatment was collected from extrinsic vein using a 5 mL sterilized syringe. Serum was collected after centrifugation and stored at −20 °C until analyses. The concentration of total cholesterol (TC) and triglycerides (TG) were measured by colorimetry using a biochemical analyzer (HITACHI 7600, Tokyo, Japan). High density lipoprotein-cholesterol (HDL-C) was measured using an HDL diagnostic kit (HDL-cholesterol kit, Youngdong Medical Corporation, Seoul, Korea). The sum of low density lipoprotein- and very-low density lipoprotein-cholesterol (LDL- + VLDL-cholesterol) was calculated by subtracting the HDL-C content from the TC.

### 2.7. Egg Quality during Storage

Four eggs were randomly selected from each replicate to observe the changes in HU during storage at 18 °C for 4 weeks. The thiobarbituric acid reactive substances (TBARS) were measured in egg yolk after 4 weeks of storage using the lipid peroxidation assay in egg yolk as described by Botsoglou et al. [21] with some modifications. In addition, five intact eggs from each treatment were stored at room temperature for 4 weeks and then egg yolk was removed and stored for 2 more weeks to study the oxidative changes in eggs. Of the collected yolk, 1.5 g was mixed with 5% aqueous trichloroacetic acid solution containing 0.8% butylated hydroxytoluene and homogenized at 4000 rpm for 5 min. After homogenization, it was reacted with thiobarbituric acid reagent and the mixture was incubated at 70 °C for 30 min. After cooling to room temperature, the absorbance of the mixture was measured at 532 nm against a blank reaction mixture. The concentration of TBARS was calculated using malondialdehyde (MDA) as a reference standard.

### 2.8. Statistical Analysis

Data were expressed as mean and the standard error of the mean (SEM). Individual cage was treated as the experimental unit for productivity- and egg quality-related parameter analyses. The experimental unit for blood parameters was an individual bird. The data of lipid oxidation in egg yolk during storage was analyzed by considering egg as an experimental unit. Orthogonal polynomial contrasts were employed to measure the linear and quadratic effects of additive dosage of FPNE using the SAS software (SAS Institute Inc., Cary, NC, USA). Differences were considered significant at *p* < 0.05. 

## 3. Results

### 3.1. Characterization of FPNE

The pH of FPNE was measured as 3.15 ± 0.02. Table 3 present the antioxidant activity and antioxidant compounds of FPNE. The contents of polyphenols and flavonoids in FPNE were determined as 50.1 ± 0.005 µg of QE/mL and 16.1 ± 0.010 µg QE/mL, respectively. The antioxidant capacity as determined by DPPH radical scavenging assay was found to be 66.9 ± 1.21%.

### 3.2. Egg Productivity

Table 4 illustrates the effect of dietary FPNE supplementation on performance of the laying hens over 6 week period. Egg production rate was significantly increased at 43 week (linear, *p* < 0.05), 45 week (linear, *p* < 0.01), and during the overall period (43–48 week) (linear, *p* < 0.01 and quadratic, *p* < 0.05) with the increasing levels of FPNE in laying hens’ diet. Dietary FPNE significantly improved the feed intake linearly during 44–45 week (linear, *p* < 0.05) and the entire period (43–48 week) (linear, *p* < 0.05). In addition, egg mass was significantly increased in FPNE supplemented groups at 45 week (linear, *p* < 0.01) and for the overall period (linear, *p* < 0.05). However, supplementing the diet with FPNE did not affect the egg weight and FCR at any time of the experimental period (*p* > 0.05).

### 3.3. Egg Quality

Table 5 shows the effect of supplemental FPNE on egg quality traits in hens from 43 to 48 week. The egg quality data showed high variability at each week of experiment. For instance, HU was significantly decreased at 46 week (linear, *p* < 0.001 and quadratic, *p* < 0.01) by the dietary supplementation of FPNE. However, no significant effect was observed at 43, 44, 46, 47, and 48 week (*p* > 0.05). Eggshell color was significantly increased in FPNE supplemented groups at 44 week (quadratic, *p* < 0.01) and 45 week (linear, *p* < 0.01). However, no significant effects were observed in egg yolk color scores among the dietary treatments at each week of study (*p* > 0.05). The eggshell breaking strength was significantly improved in the FPNE fed groups as compared with the CON group at 45 week (quadratic, *p* < 0.05), 47 week (linear, *p* < 0.01), and 48 week (quadratic, *p* < 0.05). Moreover, eggshell thickness was significantly decreased at 45 week (linear, *p* < 0.05) while significantly increased at 48 week (quadratic, *p* < 0.05) by dietary FPNE. Considering the overall effect of FPNE supplementation for 6 week, eggshell color (linear, *p* < 0.01), egg yolk color (quadratic, *p* < 0.01), and eggshell breaking strength (linear, *p* < 0.05 and quadratic, *p* < 0.05) were significantly increased in FPNE-supplemented groups. However, there was no significant difference in eggshell thickness among the dietary groups (*p* > 0.05) during 43–48 week. Furthermore, dietary FPNE negatively affected the HU (quadratic, *p* < 0.05) during the entire experimental period.

### 3.4. Blood Lipid Profile

Data of serum lipid parameters at the end of the experiment (48 week of age) are presented in Table 6. The addition of FPNE to laying hens’ diet had no significant effect on serum lipid parameters (*p* > 0.05).

### 3.5. Effect of FPNE Supplementation on Haugh Unit and Lipid Oxidation of Eggs during Storage

The effect of dietary FPNE supplementation on the egg quality during storage is presented in the Table 7. The HU decreased with the increasing storage period at 18 °C among all the treatments. Dietary FPNE did not affect the HU of eggs during storage when compared with the non-supplemented group. However, supplementation of laying hens’ diet with FPNE significantly decreased egg yolk MDA concentrations (linear, *p* < 0.001 and quadratic, *p* < 0.05) after 6 weeks of storage, as compared with the control group.

## 4. Discussion

In this study, we aimed to evaluate the potential effects of supplementing FPNE to layers’ diet on productivity performance, egg quality, serum lipid parameters, and antioxidant capacity of eggs. The addition of FPNE to laying hens’ diet increased the overall egg production and egg mass as compared with the control group. It could be possible that FPNE might have stimulated the hepatic secretion of egg yolk precursors through protecting hepatocytes from oxidative damage resulting in the enhancement of yolk formation and ovulation [22,23]. The increased egg mass in this study was related to the improved egg production rate in FPNE-fed hens. In contrast, recently, Moon et al. [24] indicated that FPNE (0.4 and 1.2 mL/kg of diet) as a component of PFA mixture had no effects on egg production and egg mass when averaged for 6 weeks. This variability in result might be related to the lower dose of FPNE or multiple components present in the feed additive mixture. In line with the present study, many studies of the inclusion of phytogenic preparations in layers’ diets indicated the dose-dependent increase in the feed intake in hens [24,25]. It is generally considered that PFA could improve the flavor and palatability of poultry feed, subsequently increasing the total feed intake. The observed improvements in egg production, egg mass, and feed intake with FPNE supplementation might be due to the presence of essential oils, terpenoids, and polyphenols which are reported to improve digestion, absorption, and utilization of nutrients in the digestive tract [3,5]. A dietary supplementation level of 0.5 mL/kg FPNE in the layers’ diet resulted in the best improvement in productive performance in terms of egg laying rate, egg mass, and feed intake.

The current results indicate that FPNE can be used to enhance the intensity of the egg yolk and eggshell color as well as the breaking strength of the eggshell. In accordance with results from the present study, increased yolk pigmentation was found by several authors when supplementing PFA to laying hens’ diet [5,23]. The effect of FPNE supplementation in laying hen diets on egg yolk and eggshell color, as well as eggshell breaking strength, was not reported previously. The higher egg yolk color score in FPNE supplemented groups could be attributed to the antioxidant components of FPNE which might have reduced the lipid peroxidation in egg yolk. Moreno and Osorno [26] reported that antioxidant components are responsible for eggshell color; however, the effect of dietary additives was not considered by the authors. The higher eggshell breaking strength in the eggs from FPNE-fed hens could be explained by the study of Radwan Nadia et al. [27], who reported that better eggshell breaking strength in the phytogenic supplemented group might be due to the fact that natural antioxidant compounds promote uterine health and increase calcium absorption as well as improve digestibility of nutrients. However, the exact mechanism is not completely understood. HU is the measure of internal egg quality. Generally, eggs are graded according to their HU values: AA, ≥72; A, 71–60; and B, <60 [24]. Herein, HU in all the dietary treatments were above 80, suggesting that the eggs produced in this study are of good quality. The internal quality of eggs deteriorated as the storage time increased, especially at room temperature [28]. The dietary inclusion of FPNE reduced egg yolk MDA concentrations during storage for 6 weeks, suggesting its antioxidant action through reducing lipid peroxidation. A dietary supplementation level of 0.25 mL/kg FPNE in the layers’ diet quadratically improved some of the egg quality parameters such as egg yolk color, eggshell breaking strength, and egg yolk antioxidant capacity. Since different concentrations of FPNE resulted in the higher values for productivity and egg quality parameters, it is imperative to conduct additional studies for the optimization of dietary supplementation levels of FPNE in laying hens.

Pine needles have been shown to promote lipid metabolism when supplemented in poultry diets [29,30]. Cholesterol and triglycerides are the important markers of lipid metabolism. Guo et al. [29] demonstrated the reduction of serum cholesterol and triglycerides in broilers by pine needle powder supplementation (10 and 50 g/kg of diet) via improving the antioxidant functions in birds. The cholesterol-reducing effects of FPNE have also been previously reported in rats fed high cholesterol diet [15]. However, in the present study, no effect of dietary FPNE on serum lipid metabolites was observed, which may be due to its lower dose or short-term supplementation.

## 5. Conclusions

The dietary supplementation of FPNE significantly improved the egg production, egg mass, and feed intake in a dose dependent manner. The egg quality parameters like egg yolk color, eggshell color, eggshell breaking strength, and egg yolk antioxidant capacity were also increased by FPNE. Further long-term studies are needed to ascertain the effect of dietary FPNE on antioxidant capacity in laying hens.

## Figures and Tables

**Table 1:** The nutrient composition of pine needles ^1^.

Items	Amount
Moisture, %	14.2 ± 0.120
Crude protein, %	27.0 ± 0.056
Crude fat, %	4.56 ± 0.141
Crude fiber, %	11.8 ± 0.134
Crude ash, %	14.8 ± 0.084
Calcium, %	0.715 ± 0.007
Phosphorus, %	0.835 ± 0.021
Acid detergent fiber, %	15.0 ± 0.176
Neutral detergent fiber, %	19.2 ± 0.268
Potassium, ppm	46,591 ± 23.2
Magnesium, ppm	2092 ± 14.8
Sodium, ppm	362 ± 18.6
Iron, ppm	1080 ± 11.1
Sulfur, ppm	1329 ± 48.4

^1^ The results are presented as mean ± standard deviation. Percentages (%) are expressed as dry matter basis.

**Table 2:** Ingredients and nutrient composition of the basal diet.

Items	Composition (%)
**Ingredients**	
Corn	50.0
Tallow	0.900
Corn dried distillers’ grains with solubles	21.2
Soybean meal	7.42
Rapeseed meal	5.00
Sesame seed oil meal	2.00
Feather meal	1.50
Syn. Lys-sulfate	0.38
Syn. Met (liq.)	0.105
Syn. Thr	0.026
Limestone	10.20
Mono dicalcium phosphate	0.590
Salt	0.200
Sodium bicarbonate	0.100
Vitamin premix ^1^	0.110
Mineral premix ^2^	0.180
Choline-chloride (50%)	0.049
**Calculated nutrient composition**	
Crude protein, %	17.0
Crude fat, %	5.30
Crude fiber, %	3.70
Ca, %	4.00
Available P, %	0.270
Lys, %	0.830
TSAA ^3^, %	0.720
TMEn ^4^, kcal/kg	2800

^1^ Vitamin premix provided followings per kg of diet: vitamin A, 40,000 IU; vitamin D3, 8000 IU; vitamin E, 10 IU; vitamin K3, 4 mg; vitamin B1, 4 mg; vitamin B2, 12 mg; vitamin B6, 6 mg; vitamin B12, 0.02 mg; pantothenic acid, 20 mg; folic acid, 2 mg; nicotinic, 60 mg. ^2^ Mineral premix provided followings per kg of diet: Fe, 30 mg; Zn, 25 mg; Mn, 20 mg; Co, 0.15 mg; Cu, 5 mg; Se, 0.1 mg. ^3^ TSAA: Total sulfur amino acid. ^4^ TMEn: Nitrogen corrected true metabolizable energy.

**Table 3:** Antioxidant activity, total polyphenols, and total flavonoids in fermented pine needle extract ^1^.

Items	Amount
DPPH ^2^ radical scavenging activity (% inhibition)	66.9 ± 1.21
TPC (µg of QE/mL) ^3^	50.1 ± 0.005
TFC (µg of QE/mL) ^4^	16.1 ± 0.010

^1^ The results are presented as mean ± standard deviation. ^2^ DPPH: 2,2-diphenyl-1-picrylhydrazyl. ^3^ TPC: Total polyphenol content was expressed as microgram of quercetin equivalent (QE) per ml of the extract. ^4^ TFC: Total flavonoid content was expressed as microgram of quercetin equivalent (QE) per ml of the extract.

**Table 4:** Effect of dietary supplementation of fermented pine needle extract in egg production and feed conversion ratio of laying hens.

Items	Treatment ^1^			SEM ^2^	*p* Value	
	CON	T1	T2		Linear	Quadratic
week 43						
Egg production (%)	83.7	90.1	92.1	1.75	0.049	0.516
Egg weight, g	64.3	62.5	63.3	0.367	0.224	0.101
Egg mass, g/hen/day	53.8	56.3	58.2	1.03	0.098	0.895
Feed intake, g/hen/day	114	113	118	1.05	0.122	0.227
FCR ^3^, g feed/g egg	2.12	2.02	2.02	0.046	0.426	0.641
**week 44**						
Egg production (%)	87.5	94.2	94.0	1.66	0.116	0.310
Egg weight, g	63.2	62.4	63.5	0.377	0.751	0.299
Egg mass, g/hen/day	55.3	58.8	59.7	1.19	0.164	0.613
Feed intake, g/hen/day	110	118	120	1.76	0.010	0.335
FCR, g feed/g egg	1.95	2.02	2.03	0.044	0.478	0.751
**week 45**						
Egg production (%)	85.0	92.4	92.4	1.28	0.005	0.059
Egg weight, g	63.0	62.3	63.7	0.286	0.322	0.074
Egg mass, g/hen/day	53.5	57.5	58.8	0.803	0.002	0.215
Feed intake, g/hen/day	94.7	105.3	106.5	1.97	0.005	0.125
FCR, g feed/g egg	1.77	1.83	1.81	0.029	0.585	0.542
**week 46**						
Egg production (%)	88.9	95.1	93.3	1.44	0.210	0.186
Egg weight, g	63.8	63.2	62.6	0.412	0.277	0.986
Egg mass, g/hen/day	56.7	60.1	58.3	0.953	0.502	0.233
Feed intake, g/hen/day	122	121	123	0.667	0.568	0.627
FCR, g feed/g egg	2.15	2.02	2.10	0.031	0.526	0.123
**week 47**						
Egg production (%)	92.1	95.4	93.3	0.950	0.631	0.219
Egg weight, g	64.2	63.8	63.5	0.414	0.577	0.952
Egg mass, g/hen/day	59.2	60.8	59.3	0.756	0.966	0.364
Feed intake, g/hen/day	122	121	123	0.821	0.641	0.295
FCR, g feed/g egg	2.07	1.99	2.08	0.029	0.891	0.175
**week 48**						
Egg production (%)	88.0	90.1	92.4	1.25	0.186	0.965
Egg weight, g	63.9	63.3	63.1	0.313	0.360	0.818
Egg mass, g/hen/day	56.2	57.0	58.3	0.912	0.407	0.918
Feed intake, g/hen/day	113	112	115	1.03	0.607	0.359
FCR, g feed/g egg	2.03	1.97	1.97	0.026	0.573	0.674
**Overall period (week 43–48)**						
Egg production (%)	87.5	92.9	92.9	0.895	0.002	0.040
Egg weight, g	63.7	62.9	63.3	0.302	0.574	0.407
Egg mass, g/hen/day	55.8	58.4	58.8	0.610	0.042	0.316
Feed intake, g/hen/day	113	115	117	0.839	0.013	0.990
FCR, g feed/g egg	2.02	1.98	2.00	0.019	0.831	0.511

^1^ CON: basal diet; T1: basal diet + 0.25 mL/kg of fermented pine needle extract; T2: basal diet + 0.5 mL/kg of fermented pine needle extract. ^2^ SEM, Standard error of the mean. ^3^ FCR: feed conversion ratio, measure of an animal’s efficiency in converting feed mass into increases of the desired output.

**Table 5:** Supplementary effect of fermented pine needle extract in egg quality of laying hens.

Items	Treatment ^1^			SEM ^2^	*p* Value	
	CON	T1	T2		Linear	Quadratic
week 43						
Haugh unit	97.0	92.8	92.5	1.36	0.189	0.497
Eggshell color	10.07	10.50	10.37	0.169	0.497	0.460
Egg yolk color	6.83	7.37	7.10	0.120	0.358	0.124
ESBS ^3^, kg/cm^2^	3.04	3.56	3.25	0.199	0.461	0.110
EST ^4^, mm	0.327	0.333	0.327	0.004	0.998	0.497
**week 44**						
Haugh unit	92.4	91.5	93.3	0.788	0.678	0.459
Eggshell color	10.5	11.6	11.1	0.152	0.056	0.007
Egg yolk color	6.67	7.20	6.77	0.118	0.713	0.057
ESBS, kg/cm^2^	3.20	3.62	3.62	0.092	0.058	0.249
EST, mm	0.371	0.364	0.363	0.005	0.528	0.807
**week 45**						
Haugh unit	90.9	93.9	94.8	1.01	0.140	0.633
Eggshell color	9.93	11.3	11.7	0.253	0.001	0.187
Egg yolk color	7.07	7.17	7.20	0.095	0.600	0.879
ESBS, kg/cm^2^	3.57	3.92	3.51	0.082	0.749	0.026
EST, mm	0.377	0.373	0.357	0.004	0.037	0.430
**week 46**						
Haugh unit	97.2	84.7	85.3	1.74	< 0.001	0.005
Eggshell color	11.6	11.8	11.5	0.129	0.770	0.539
Egg yolk color	7.53	7.73	7.47	0.097	0.790	0.293
ESBS, kg/cm^2^	3.27	3.19	3.01	0.093	0.296	0.816
EST, mm	0.358	0.351	0.354	0.003	0.582	0.459
**week 47**						
Haugh unit	98.6	95.8	98.6	0.970	0.983	0.205
Eggshell color	11.3	11.4	11.7	0.157	0.335	0.678
Egg yolk color	7.40	7.83	7.67	0.088	0.197	0.102
ESBS, kg/cm^2^	2.85	3.52	3.90	0.171	0.008	0.625
EST, mm	0.360	0.374	0.367	0.004	0.513	0.275
**week 48**						
Haugh unit	97.1	96.5	98.4	0.655	0.452	0.391
Eggshell color	11.0	11.4	11.6	0.156	0.132	0.685
Egg yolk color	7.53	7.33	7.47	0.116	0.828	0.534
ESBS, kg/cm^2^	2.97	3.74	3.33	0.130	0.188	0.024
EST, mm	0.363	0.384	0.375	0.004	0.151	0.046
**Overall period (week 43–48)**						
Haugh unit	95.6	92.5	93.8	0.536	0.148	0.047
Eggshell color	10.75	11.3	11.3	0.101	0.008	0.088
Egg yolk color	7.17	7.44	7.28	0.038	0.130	0.003
ESBS, kg/cm^2^	3.15	3.53	3.44	0.059	0.019	0.023
EST, mm	0.359	0.363	0.357	0.002	0.639	0.225

^1^ CON: basal diet; T1: basal diet + 0.25 mL/kg of fermented pine needle extract; T2: basal diet + 0.5 mL/kg of fermented pine needle extract. ^2^ SEM, Standard error of the mean. ^3^ ESBS: Eggshell breaking strength. ^4^ EST: Eggshell thickness.

**Table 6:** Effect of FPNE supplementation on serum lipid parameters in laying hens at 48 week of age.

Items (mg/dL)	Treatment ^1^			SEM ^2^	*p* Value	
	CON	T1	T2		Linear	Quadratic
Triglycerides	1175	1385	1239	81.4	0.756	0.322
Total cholesterol	134	145	133	5.64	0.986	0.375
HDL ^3^-cholesterol	10.22	9.72	9.64	0.545	0.682	0.867
VLDL + LDL ^4^-cholesterol	123	133	124	5.30	0.981	0.461

^1^ CON: basal diet; T1: basal diet + 0.25 mL/kg of fermented pine needle extract; T2: basal diet + 0.5 mL/kg of fermented pine needle extract. ^2^ SEM, Standard error of the mean. ^3^ HDL: high density lipoprotein. ^4^ VLDL + LDL-cholesterol: value calculated to subtract HDL-cholesterol from the total cholesterol.

**Table 7:** Changes in Haugh unit and lipid oxidation of eggs during storage.

Items	Period	Treatment ^1^			SEM ^2^	*p* Value	
		CON	T1	T2		Linear	Quadratic
Haugh unit	week 1	87.1	82.5	84.4	1.28	0.399	0.258
	week 2	72.6	71.3	72.1	0.538	0.698	0.409
	week 3	63.5	61.9	64.1	0.827	0.763	0.310
	week 4	60.9	60.5	59.6	1.45	0.741	0.940
MDA, μg/g yolk ^3^	week 4	0.013	0.014	0.015	0.001	0.156	0.702
MDA, μg/g yolk ^4^	week 6	0.069	0.019	0.018	0.008	<0.001	0.022

^1^ CON: basal diet; T1: basal diet + 0.25 mL/kg of fermented pine needle extract; T2: basal diet + 0.5 mL/kg of fermented pine needle extract. ^2^ SEM, Standard error of the mean. ^3^ MDA: Malondialdehyde measured in eggs stored for 4 weeks. ^4^ MDA: Malondialdehyde measured in eggs stored for 4 weeks and with open-contamination condition without eggshell for 2 more weeks.

## Data Availability

The data are available on request from the corresponding author.
